# Manual therapy applied by general practitioners for nonspecific low back pain: results of the ManRück pilot-study

**DOI:** 10.1186/s12998-018-0202-2

**Published:** 2018-09-03

**Authors:** Heidrun Lingner, Lena Blase, Anika Großhennig, Guido Schmiemann

**Affiliations:** 1 Centre for Public Health and Healthcare, Hannover Medical School, Carl- Neuberg-Straße 1, 30625 Hannover, Germany; 2 Institute of Biostatistics, Hannover Medical School, Hannover, Germany; 3 Institute for General Practice, Hannover Medical School, Hannover, Germany; Department for Health Services Research, Institute for Public Health and Nursing Science, University of Bremen, Grazer Str. 4, 28359 Bremen, Germany

**Keywords:** Manual therapy, Low back pain, General practice, Primary care

## Abstract

**Background:**

Nonspecific acute low back pain (LBP) is a common reason for accessing primary care. German guidelines recommend non-steroidal anti-inflammatory drugs and physical activity as evidence-based treatments. Manual Therapy (MT) remains controversial. To increase evidence-based treatment options for general practitioners (GPs), a Pilot-Study was set up to gather information about the required conditions and setting for an RCT.

**Methods:**

The open pilot-study assesses recruitment methods for GPs and patients, timelines, data collection and outcomes of treatment immediately (T0) and 1, 6 and 12 weeks after consultation (T1, T2, T3). Inclusion criteria for GPs were: no experience of MT; for patients: adults between 18 and 50 suffering from LBP for less than 14 days.

Study process: Patients’ control-group (CG) was consecutively recruited first and received standard care. After GPs received a single training session in MT lasting two and a half hours, they consecutively recruited patients with LBP to the intervention group (IG). These patients received add-on MT.

Primary outcomes: (A): timelines and recruitment success, (B): assessment tools and sample size evaluation, (C) clinical findings: pain intensity change from baseline to day 3 and time till (a) analgesic use stopped and (b) 2-point pain reduction on an 11-point scale occurred.

Secondary outcomes: functional capacity, referral rate, use of other therapies, sick leave, patient satisfaction.

**Results:**

14 GPs participated, recruiting 42 patients for the CG and 45 for the IG; 49% (56%) of patients were women. Average baseline pain was 5.98 points, SD: ±2.3 (5.98, SD ±1.8).

For an RCT an extended timeline and enhanced recruitment procedures are required. The assessment tools seem appropriate and provided relevant findings: additional MT led to faster pain reduction. IG showed reduced analgesic use and reduced pain at T1 and improved functional capacity by T2.

**Conclusions:**

Before verifying the encouraging findings that additional MT may lead to faster pain reduction and reduced analgesic use via an RCT, the setting, patients’ structure, and inclusion criteria should be considered more closely.

**Trial registration:**

Number: DRKS00003240 Registry: German Clinical Trials Registry (DRKS) URL: https://www.drks.de/drks_web/. Registration date: 14.11.2011. First patient: March 2012. Funding: the Rut and Klaus Bahlsen Stiftung, Hannover.

## Background

Acute nonspecific low back pain (LBP) with a one-year prevalence of up to 76% is a major health problem and a common reason for consulting a general practitioner (GP) [1, 2]. The high socioeconomic burden caused by the direct and indirect costs of LBP has often been described [3, 4]. More importantly, patients’ quality of life is markedly reduced. Nevertheless, GPs have few evidence-based treatment options [5]. National and international guidelines propose prescription of analgesics and recommend that patients rapidly resume activity and avoid bed rest [6, 7]. Although multiple clinical practice guidelines include manual therapy (MT) as a therapeutic option, the results of heterogeneous MT-trials [8–11] are inconsistent. MT, defined as a general, spinal manipulative manual therapy [11] using low-velocity mobilization and/or high-velocity manipulation techniques [12–14] is therefore currently not strongly advocated for LBP treatment [5].

An American study focusing on MT provided by GPs, however, showed some small but encouraging results, particularly on the improvement of patients’ physical function [15]. The GPs were experienced in MT and recruited/ treated the intervention and the control group in parallel. This may have created a selection and treatment bias leading to an underestimation of the benefits of MT, especially in pain reduction. Facing the high prevalence of acute, non-specific LBP, the frequency of related consultations, the high rate of analgesic use and the lack of evidence-based non-pharmaceutical options to treat LBP in Germany, the ManRück pilot trial (Manuelle Therapie bei unspezifischen akuten Rückenschmerzen – manual therapy in unspecific acute back pain) investigated whether the projected study concept is feasible as planned, in preparation for an RCT, by answering the following questions:

- Are the intended recruitment procedures and proposed supporting strategies sufficient?
- Are the tools chosen for the GPs’ examinations and treatment and the patients’ feedback appropriate for use in this field of research?
- Is there evidence, such as would support running an RCT, that the MT-techniques used here and provided by MT naïve general practitioners to patients with nonspecific LBP lead to faster pain reduction compared to standard care?

## Methods

The ManRück pilot trial is a non-blinded, multicentre intervention study to assess the feasibility of a randomized control trial (RCT) which will quantify the impact of standardized MT on patients with LBP. The rationale for the study design is detailed in the study protocol and has been described in depth elsewhere [15].

**Fig. 1:**
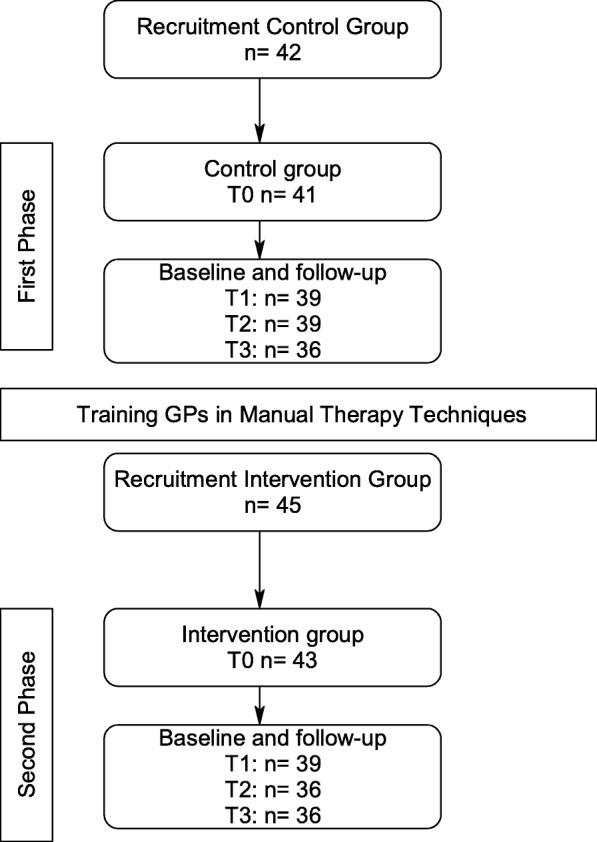
Patients’ recruitment and flowchart of ManRück

The study progress is shown in Fig. 1. GPs were required to be unfamiliar with MT to participate in the ManRück study. When the GPs obtained written informed consent, they first began to recruit participants for the control group (CG). These consecutively recruited eligible patients received usual care from their GPs. Following a training session in MT for LBP by an expert GP with an additional qualification in chiropractic and a teaching accreditation from the Medical Association of Lower Saxony, the same GPs who had recruited CG patients and provided usual care then recruited patients for the intervention group (IG).

**Fig. 2:**
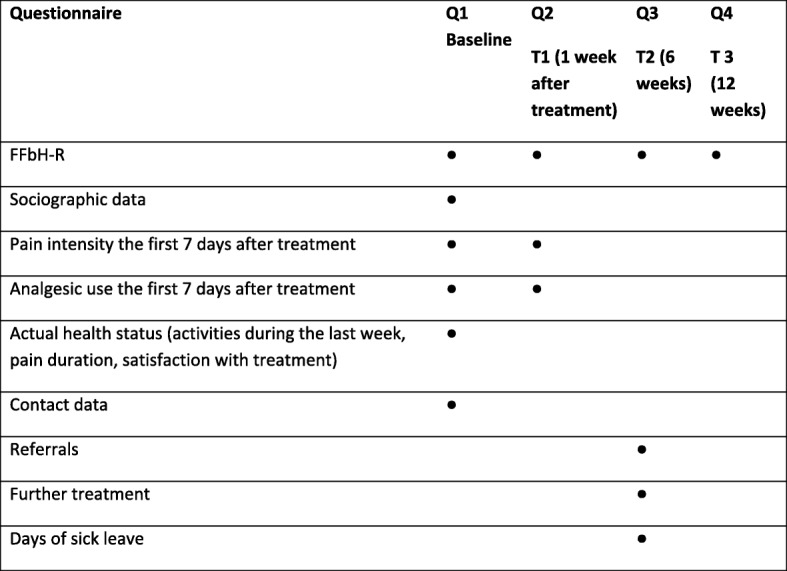
Distributed documents, content of questionnaires Q1-Q4 and timetable of data collection

These patients received MT and usual additional care if it was required. The treatment process and the study end-points were documented by questionnaires and pain diaries at T0 (baseline), and after 1, 6 and 12 weeks (T1-T3) (Fig. 2). Additionally, IG and CG-patients who did not answer the questionnaire on time were reminded of their study participation once by telephone. All data collected by diary and questionnaires at T2-T4 were also collected by phone interviews.

Because the pilot study aimed to gather useful information to avoid pitfalls when running the subsequent RCT, the targeted RCT-outcomes had to be adjusted to include additional queries as follows:

### Primary outcomes

A. **Combined for ManRück pilot and intended RCT:** (1) change in patients’ self-reported pain perception from T0 to day 3, measured by an 11-point combined visual analogue and numeric rating scale designed and recommended by the German Pain Society (VAS-NRS) and (2) a combined outcome of (a) time from T0 until a reduction of 2 points on the VAS-NRS occurred and b) time till patients stopped using analgesic drugs.
B. **For the ManRück pilot:** (1) time till 20 GPs consented to participate, (2) number of enrolled CG and IG patients over a 2 week time period, (3) completeness of pain diary and questionnaires compared to the data gathered by phone interviews and (4) number of returned diaries and questionnaires.

### Secondary outcomes

A. **Combined for ManRück pilot and the intended RCT:** (1) change in value of pain intensity on the VAS-NRS from T0 to day 7 (T1), (2) self-reported amount of analgesic use (prescribed or over the counter), (3) functional capacity assessed by the Funktionsfragebogen-Rücken (Hannover functional ability questionnaire – spine (FFbH-R), (4) referral by the GP for further treatment (yes/no), (5) use of other forms of therapy, (6) duration of sick leave (days) and (7) patient satisfaction with their treatment recorded at T0 using a Likert scale (0–10).
B. **For the ManRück pilot:** assess the feasibility of teaching specific MT techniques to MT naïve GPs during a single 2 ½ hour training session.

The FFbH-R questionnaire consists of 12 items describing activities of daily life and the patients’ ability to perform these on a three-point scale (possible without problems, possible with problems, impossible), with a higher score indicating a better functional capacity.

The timetable of the data collection and the content of the distributed questionnaires Q1-Q4 are shown in Fig. 2. CG and IG patients received the same study documentation consisting of 4 questionnaires (Q1-Q4), a VAS/NRS and a patient’s diary (PD) to document the intensity of pain and the use of analgesics on a daily basis, starting upon inclusion in the study and during the first 7 days following treatment. For detailed information on the data collection see Fig. 2. Additionally, all patients from both study groups were advised to consult their GP immediately if pain increased or any other unexpected or concerning events occurred. Selected items of the Orebro questionnaire [16] addressing the presence of risk factors for chronification were used to assess symptoms and severity during the week preceding the GP consultation.

The training for the GPs in standardized MT suitable for the treatment of LBP was planned and agreed upon by an expert panel drawn from the different German MT schools [17, 18]. Due to the study design, the GPs were aware of the two recruiting phases for CG and IG. The consecutively recruited patients, however, were “blind” regarding their allocation to CG or IG, as this depended solely on the point in time that they appeared in the surgery. They had no knowledge of the newly acquired MT-skills of their GP.

#### Participating GPs

Practicing GPs from teaching practices associated with the Hannover Medical School and from the registry of the Association of Statutory Health Insurance of Lower Saxony were invited by telephone to participate in the study. The lists of both groups were sequentially followed until 20 GPs gave their written informed consent to participation. Exclusion criteria were: previous training in chiropractic or experience in osteopathy or physical therapy. An incentive of 100€ was not advertised during recruitment but assigned during the trial at the end of the CG treatment phase.

#### Participating patients

All patients meeting the inclusion criteria (Table 1) were invited to participate in the study by their GP.

**Table 1:** Inclusion and Exclusion criteria of LBP-patients

Inclusion criteria	Exclusion criteria
age 18 to 50	diseases of the spine (e.g., osteoporosis or rheumatic diseases)
acute (≤14 days), non-traumatic and non-specific LBP	spine surgery during the last 6 months
Localisation: between the costal margin and the gluteus folds	fracture, radiculopathy, cauda equina syndrome, signs of infection, signs of tumor of the spine
written informed consent	Pregnancy
	ongoing treatment by a chiropractor or physiotherapist
	any other treatment for LBP during the last 6 months
	poor language or reading skills

Patients younger than 18 were excluded as their inclusion would require additional parental approval. We limited the participants’ age to 50 and below, as the risk of LBP caused by specific illnesses increases with age [19].

### Interventions

Participating GPs were required to carefully examine the patients and to use the appropriate documentation provided and the predefined expert-approved approach for clinical examination and history taking [17], as summarized in Table 2.

**Table 2:** General overview of examination for LBP-patients [1] used in ManRück

History taking:	
● duration of pain	
● loss of muscle power	
● loss of sensitivity	
● fever	
● traumatic injury	
● known osteoporosis	
● weight-loss, night-sweats or other signs of cancer	
Clinical examination	
The examination should be conducted with the patient standing without shoes. If possible, the patient should also wear no trousers and be examined with a free lower back from rib cage to the posterior superior iliac spine.	
Examination while the patient is standing:	
● Inspection of the spine (faulty posture/signs of traumatic injury)	
● Indication by the patient of a) pain localization and b) radiation where applicable	
● Walking on tiptoes and on the heels (to identify damage to S1 and L5)	
● Spine test	
● Standing forward flexion test	
Examination while the patient is sitting:	
● Quadriceps test (patient straightens leg against the examiner’s hand)	
Examination while the patient is lying:	
● Sensitivity in both legs	
● Characteristic muscles for L2/3 - > Adduction	
● Characteristic muscle for L5 - > M. ext. hallucis long.	
● Lasègue- and Bragard’s test	
● The flexibility of the hip joints (interior and exterior rotation)	

#### Usual care

Analgesics and other forms of therapy (e.g., physiotherapy) were prescribed by the GPs according to their usual treatment methods. There were no restrictions on their usual criteria for referral of patients to other professionals as they found appropriate.

#### Manual therapy techniques

An expert panel discussed, selected and agreed on techniques to be taught to the MT-inexperienced GPs. High-velocity, low-amplitude (HVLA) thrusts were excluded for safety reasons. Moreover, only techniques that could be taught within 2.5 h were eligible. Two diagnostic tests and three therapeutic techniques focusing on the sacroiliac joint (SIJ) and the lumbar spine were chosen: see Table 3.

**Table 3:** MT techniques used in the ManRück study [17]

Diagnostic tests	**Spine-Test:** The GP places his thumbs at an equal level, one on the posterior superior iliac spine (PSIS) and the other on the Os sacrum. The patient lifts a leg maximally. If the therapist’s thumb on SIPS does not slide caudal, the test is positive for SIJ-dysfunction [27].
	**Standing forward flexion-Test:** The GP is standing behind the standing patient and places his thumbs on both PSIS. The patient bends forward. If one PSIS moves further forward, the test is positive for SIJ-dysfunction [37].
Manual therapeutic techniques	**1.Vibrating traction of both legs**: the patient is either lying on the back or face down, the GP takes hold of the legs above the ankle with both hands and pulls the legs vibrating simultaneously [37, 38]. Vibrating traction was presented with a low frequency (20–40/min) at an angle between 20 and 40°. The traction was applied for roughly 1–3 min without a specified duration.
	**2. Post-isometric relaxation**: Patient on the back with bent knees. The patient abducts both legs against the resistance of GPs’ hands. The same exercise is then performed with adduction in the hip. Both maneuvers were completed once for 15 s.
	**3. The mobilization of the iliosacral joint and the lumbar spine**: The patient lies on his side with his upper leg bent 90 degrees at the hip. The GP stands in front of the patient and places the hand closest to the patient’s head on the patient’s upper shoulder. The GP’s other hand is placed on the patient’s hip. By moving the shoulder and the upper leg of the patient, the GP can adjust the rotation of the spinal column. The stretch is held for a few seconds and then released. The other side of the back is treated in the same way [12].

### Data collection

Due to the high prevalence of LBP, we expected 20 GPs to be able to successfully recruit at least 10 patients to the CG during a period of 2 weeks.

At the baseline visit (T0) patients completed the first part of Q1 (incl. 12 items of the FFbH-R) before seeing the GP. The second part of Q1, including general health-related and pain-specific questions as well as items referring to patient satisfaction regarding the quality of care provided by the GP, was answered in the practice immediately after the consultation. Although some of these questions could have been addressed before seeing the doctor, we tried to keep the first part short to minimize disturbance of the consultation proceedings.

Intensity and perception of pain and its consequences (sick leave) during the previous week were documented using selected questions from the Örebro Musculoskeletal Pain Screening Questionnaire (OMPSQ) [1]. This also helped to assess the risk for chronification of the LBP symptoms. Additionally, sociodemographic information on age, sex, academic education and employment status was collected at the end of the questionnaire.

Q1 was sent by post to the study center by the patients in a sealed, prepared envelope. Questionnaires Q2-Q4 and patient diaries were completed at home and also sent by post to the study center. To validate the answers and minimize data loss, patients’ answers were additionally collected by phone interviews at T2-T4 during the evening hours on the respective days. If the participant could not be reached, the call was repeated the following day. In case of discrepancies, results of the telephone interview were used to analyze the clinical outcomes.

The diaries documented self-assessed pain severity (using an 11-point combined VAS-NRS) and analgesic intake from T0-T1. The patients marked their pain intensity on a continuum between a sad and a smiling face. An 11-point NRS was printed on the reverse, and after indicating the appropriate level, the patients turned the scale over to read off the numerical value corresponding to their mark. A change of 2 points on the NRS was accepted as the minimal clinically relevant difference [20]. The combined VAS-NRS was used to assign numerical values to the perceived pain intensity. This allowed us to compare the pain values at different time points for the same patient and also between the groups.

The FFbH-R (distributed at T0-T3) assesses the subjective functional ability to perform daily activities on a 0–100% scale using 12 questions. The minimal clinically relevant difference is estimated to be 12% [21].

#### Sample size

The sample size of patients was estimated according to the requirements of the clinical primary and secondary outcomes. The intervention was considered globally superior if (a) the change in numeric pain scale on day 3 is non-inferior compared to the change that is achieved by standard therapy and (b) the second composite end point (time until pain perception is reduced by 2 points on the NRS and painkiller use is stopped) is improved after the intervention. Our non-inferiority margin was defined as an improvement of 1 visual analog scale point.

For the first primary outcome, a sample size of 86 patients per group was necessary to show the non-inferiority of additional MT compared to usual care with a power of 90% (Query Advisor®, Version 7.0). Due to the lack of published appropriate and defined assumptions, the same sample size was also used for the second primary endpoint. We, therefore, expected that sufficient patients would be recruited if around 10 GPs each enrolled approximately 10 patients in each group [17].

#### Statistical analysis

The primary analysis was performed using the intention to treat population (ITT). The results were compared with those of the per protocol population (PP). When data for a patient in the ITT population was incomplete, the last known VAS-NRS value was carried forward. Missing values in the PP population were not replaced.

First, analysis of covariance for the change in VAS-NRS score from T0 to day 3 was carried out, adjusted by baseline value, GP’s practice and treatment group as independent variables. Then time taken to achieve the combined outcome of VAS-NRS-reduction of at least 2 points and cessation of analgesic use was determined. We used a common Wilcoxon/Breslow-test to compare the spread of times for both groups. A sensitivity analysis adjusted for IG and GP’s practice was carried out by a Cox-regression. The median event-times and their 95%-CI were reported.

A secondary analysis used PP population with no input of missing data. The frequencies of secondary outcomes were compared for both groups by χ^2^-Test, Fisher’s exact test or Student’s t-test. Descriptive *p*-values were reported. Duration of sick leave and results of the FFbH-R questionnaire are presented in an explorative manner. For the FFBH-R a covariance analysis was used with treatment group, GP’s practice and baseline-value as independent variables and the changes from T0 to T1 and T2 as dependent variables.

Further details of the tests employed were previously published in the study protocol [17]. Statistical analysis was performed using SPSS Version 21.

## Results

### Participants’ flow chart and numbers

Overall, 124 GPs from the combined list of teaching practices associated with the Hannover Medical School and from the registry of the Association of Statutory Health Insurance of Lower Saxony practicing within the city of Hannover were eligible to participate in the study. Seventy-eight of these GPs were contacted by telephone, 50 of whom wished to receive further information. Twenty GPs provided signed written informed consent and were enrolled in the study. The overall time for the GPs’ recruitment (from first phone contact to received informed consent) was 3 months.

Starting on 16th of March 2012 the GPs recruited 42 patients to the CG. Despite their written commitment to participate and the offered encouragement, six out of the 20 GPs failed to recruit at least one patient for the CG during 1 year and therefore had to be excluded from the second recruitment phase (for IG-patients) due to the study design. Reasons given upon request were work overload and lack of time. To compensate for this high drop-out rate three further suitable GPs were contacted but finally only one participated in the study.

The second phase of the study started on 18th of February 2013, during which the GPs recruited 45 patients to the IG (see Fig. 1).

The planned total sample size could not be reached.

In total 11 patients were inaccurately included (IG *n* = 9, CG *n* = 2), as they had suffered back pain for more than 14 days, were older than 50 years or were pregnant. Nevertheless, these patients showed no significant differences in baseline data concerning pain intensity, functional capacity and the overall superiority of the add-on MT-treatment. Following the ITT approach, data for these 11 patients were included in all analyses. T0 data for 3 patients went missing. The number of analyzed answers per question (denominator) differs as some of the returned questionnaires were incomplete.

There was a high rate (*N* = 87) of returned FFbH-R questionnaires overall: 97% at baseline, 90% at T1, 86% at T2 and 83% at T3.

### Baseline characteristics

Seven male and seven female GPs with an average age of 51.6 years participated; 5 GPs worked in individual practices and 9 in group practices. Patients’ baseline characteristics are detailed in Table 4.

**Table 4:** Patient’s baseline characteristics

Baseline-Data		N	CG N(%)	IG N (%)	p-value (χ^2^-Test)
Gender, female		84	20(48.8)	24(55.8)	0.519
Family status		84			0.162
	Single		19(46.3)	19(44.2)	
	Married		15(36.6)	21(48.8)	
	Divorced/separated		7(17.1)	2(4.7)	
	Widowed		0(0)	1(2.3)	
Community/ sheltered living		84	24(58.5)	30(69.8)	0.283
School qualifications		84			0.936
	None		1(2.4)	0(0)	
	general secondary school (Haupt-/Volksschule)		4(9.8)	4(9.3)	
	Intermediate secondary school (Realschule/Mittlere Reife)		19(46.3)	18(41.9)	
	technical college entry level (Fachhochschulreife)		4(9.8)	4(9.3)	
	University entry level (allg. Hochschulreife)		13(31.7)	17(39.5)	
Employment		84			0.956
	Full time		25(61.0)	28(65.1)	
	Part-time		9(22.0)	8(18.6)	
	Occasionally employed		2(4.9)	2(4.7)	
	Seeking work		1(2.4)	0(0)	
	Retired		0(0)	0(0)	
	other (home maker, training, assisting family member)		4(9.8)	5(11.6)	
		N	Mean (±SD)	Mean (±SD)	p-value (Student‘s t-Test)
Age, years		84	38.39(9.43)	36.93(9.78)	0.488
State of health (0–10)		84	4.15(2.45)	3,14(2.37)	0.059
Pain intensity(VAS 0–10)		83	5.98(2.26)	5.98(1.76)	0.999
Functional ability (FFbHR, 0–100%)		84	53.35(22.54)	49.96(22.72)	0.494
Days of pain-duration (without interruption)		81	4.15(3.41)	6.95(11.66)	0.143
Items based on the Orebro Musculoskeletal Pain Screening Questionnaire (ÖMPSQ)^a^	Mean pain-intensity during the past week (0–10), (0 = no pain)	84	4.27(2.75)	3.79(2.73)	0.427
	Have felt tense and anxious in the past week (0–10) (0 = absolutely calm and relaxed)	83	3.80(2.59)	4.12(2.47)	0.573
	Increase of pain is an indication to stop until the pain decreases (0–10) (0 = completely disagree)	84	6.12(3.18)	6.98(2.53)	0.178
	With present pain, should not do normal work (0 = completely agree)	84	4.61(3.61)	4.14(3.54)	0.548
	Can do light work for an hour (0 = completely agree)	84	2.68(3.19)	2.26(2.32)	0.487
	Can work for an hour(0 = completely agree)	84	3.46(3.61)	2.91(2.63)	0.423
	Can do ordinary household chores (0 = completely agree)	83	4.10(3.02)	5.14(2.79)	0.105
	Can do the weekly shopping(0 = completely agree)	84	4.22(3.37)	5.58(3.21)	0.061
Patient’s satisfaction	Fast Symptom-relief by the GP (0 = completely agree)	83	5.33(4.14)	4.14(3.33)	0.156
	Exhaustive examination by GP(0 = completely agree)	83	1.08(1.98)	0.88(1.55)	0.624
	GP answered questions carefully(0 = completely agree)	83	0.80(1.24)	1.12(1.93)	0.381
	Quality of treatment GP (0 = excellent)	83	1.23(1.25)	1.58(2.13)	0.352

^a^The OMPSQ was developed to help practitioners to identify patients at risk of developing chronic back pain as early as possible

^b^Here the Fisher’s exact test was used

No statistically significant differences were detected at T0 between CG and IG, including the risk for chronic LBP, as assessed by the Orebrö questionnaire. Some of the patients (24.2% of the CG and 13.9% of the IG) reported referrals to other doctors by their GP at T2. In all cases, these referrals during the previous 6 weeks were to orthopedic specialists.

Only 76 of the 87 patients returned their pain-diaries to the study center, and the return of questionnaires decreased slowly from T0 to T3 (from *n* = 84 to *n* = 72), although remaining at a high level, whereas the data collected by phone showed only two missing entries during the whole study period. Further nonclinical findings concerning the feasibility of an RCT and influencing the deployed study-design are summed up in Table 5. Their implication is further assessed in the discussion section of this paper.

**Table 5:** Overview of outcomes regarding the feasibility of the pilot study and suggestions for improvement

Pilot study	Questions	RCT-study design relevant results	Improvement requirements and possible actions
Recruitment procedures	Time till consent obtained from 20 GPs How many patients CG/2 weeks How many patients IG/2 weeks	3 Months (expected two weeks); 6/20 GPs failed in recruitment 42 pat. in 12 months; 2pat. inaccurate 45 pat. in 4 months; 9 inaccurate	adapted to recruitment time findings Increase (at least double) the number of recruited GPs re-evaluate inclusion and exclusion criteria for patients, recruitment time and sample size of participating patients, recalculate sample size Check on included patients (fax or phone) if criteria are fulfilled; enhance recruitment in “slow” and stop “fast” recruiters when mean of correctly included pat. is reached.
Supporting/contact strategies	personal calls info. Poster non advertised 100€ incentive at the end of the CG	Monthly Seasonally adapted Advertise & split incentives for CG & IG	increase the frequency of the contact-calls to study nurses (at least weekly) more target-group adapted flyers; a higher number of seasonally adapted posters; use of “new communication channels” such as electronic messaging on screens in practice waiting rooms financial reward for the additional workload of GP, staff and possibly for the patients. Direct incentive to medical staff, “pay per capita.”
patients’ feedback tools	frequencies & lengths of data collection returned & completeness of paper-pen pain diary returned & completeness of questionnaires: OMPSQ*, FFbH-R*	adequate, but could also assess chronification 87% 90% complete data 97–83% 72% complete data	Follow up with assessments of pain intensity and painkiller intake for at least 12 weeks
Data collection	faster pain reduction compared to standard care	Yes	
	phone interviews	98% complete data	Recalculate costs (if two part-time study nurses collecting data (employed at 8%) are less expensive than prepared and prepaid envelopes, and recall-calls)
	Information about excluded pat.	Not collected!	Rework layout of documentation sheet. Explain importance in more depth and advertise allowance for this completed sheet separately
	feasibility of teaching specific MT techniques in 2 ½ hours	feasible	Assess MT quality by practice visit 2 weeks after MT-“training,” during IG

OMPSQ* Örebro Musculoskeletal Pain Screening Questionnaire

FFbH-R* Hannover functional ability questionnaire-spine

### Primary clinical outcomes

#### Pain

Both primary outcomes focused on pain reduction, measured by a combined VAS and NRS. The lower limit of the 95% confidence interval of the difference in mean pain reduction between IG and CG for the first primary outcome was above the limit of non-inferiority of − 1 in the ITT population and PP population, [ITT: (0.309 [− 0.798; 1.417]), PP: (0.593 [− 0.591; 1.776])]. The sensitivity analysis excluding the 11 patients who fulfilled the exclusion criteria showed similar results (0.240 [− 0.938; 1.418]).

**Fig. 3:**
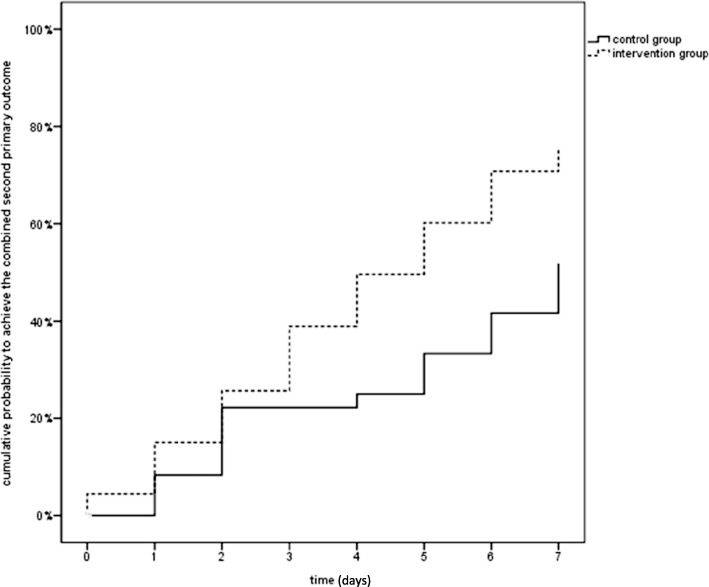
Days until the combined second primary outcome consisting of (**a**) a pain-reduction of at least 2-VAS/NRS-points and (**b**) no analgesic use was achieved

Patients in the IG reached the second primary endpoint (consisting of (a) cessation of analgesic use and (b) 2-point pain reduction on the 11-point VAS-NRS scale) earlier (see Table 6). The difference in time taken to reach this endpoint between the CG and IG, as assessed by the Wilcoxon-Breslow-test, was statistically significant (*p* = 0.021) (Fig. 3, Table 7).

**Table 6:** Primary comparison of the difference of mean pain-reduction from Baseline to day 3 at VAS/NRS (0–10) (intervention group -control group)

Population	N	Difference^a^ of mean pain-reduction (VAS)	95%-CI^a^	Standard error	*p*-value
ITT	87	0.309	−0.798,1.47	0.556	0.579
PP	74	0.593	−0.591,1.776	0.592	0.321

^a^Estimated effects of mean and 95%-confidence interval from covariance-analysis, adjusted for baseline-value, treatment group and practice as independent variables; ITT means intention-to-treat, missing values were input by last value carrying forward; PP per-protocol, only available values were included; VAS-NRS (0–10); IG, usual treatment plus add-on-manual therapy, CG, usual treatment alone

**Table 7:** Comparison of the median of days till the combined second primary outcome: pain-reduction of at least 2 VAS-points and no more use of analgesics was achieved

		Median^a^		
Therapy group	N	Estimator	95%-CI	
CG	42	7.00	.^a^	.^a^
IG	45	5.00	3.531	6.469
Total	87	6.00	4.810	7.190

^a^The CI is not available for the CG, as more than 50% of CG-patients were censored

Comparing the second combined primary outcome between the IG and CG during the first 7 days by Cox-regression, the hazard ratio was 1.961 ([1.045; 3.679] *p* = 0.036); the probability of reaching this outcome during the first week was twice as high for IG patients.

Summing up the main clinical ManRück findings; we could identify the following important indications: (1) According to our results, add-on-MT is not inferior in pain reduction from baseline to day three to usual care. (2) Moreover, add-on-MT is superior to usual care alone, as the second primary outcome (pain reduction of at least two points AND no analgesic use) was reached faster by the IG. These two facts combined and considered together indicate a potential global superiority of add-on-MT in comparison to usual care alone.

**Fig. 4:**
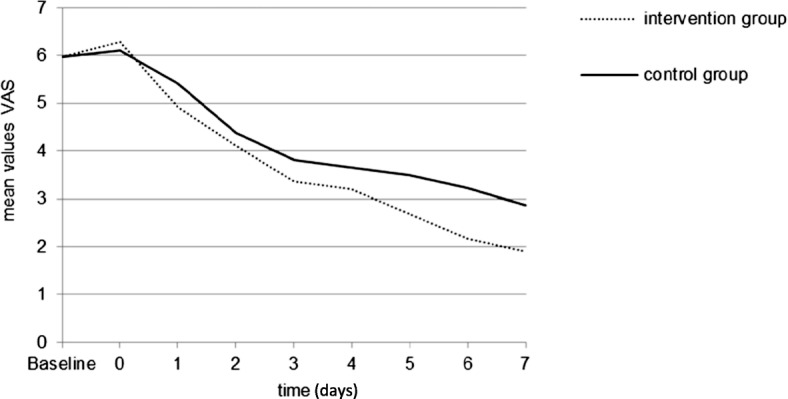
Mean values of VAS/NRS (0–10) for pain during the first week (Baseline; days 0–7) after treatment

**Fig. 5:**
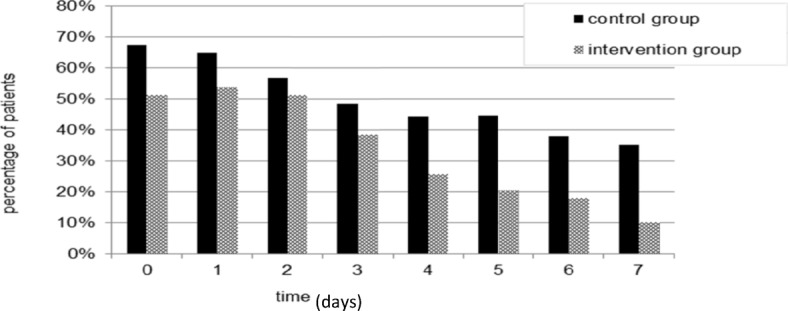
Percentage of patients consuming analgesics per day on days 0–7

From days 1 to 7, the IG patients reported less pain than patients in the CG (Fig. 4). Moreover, fewer IG patients used analgesics during the first week (Fig. 5) than those in the CG. The differences reported on days 5 and 7 were considerable (*p* = 0.023 and *p* = 0.009 respectively). As the sample size calculation focused on the power of the primary outcomes, however, this finding can only be interpreted as a tendency.

### Secondary outcomes

#### Function

**Fig. 6:**
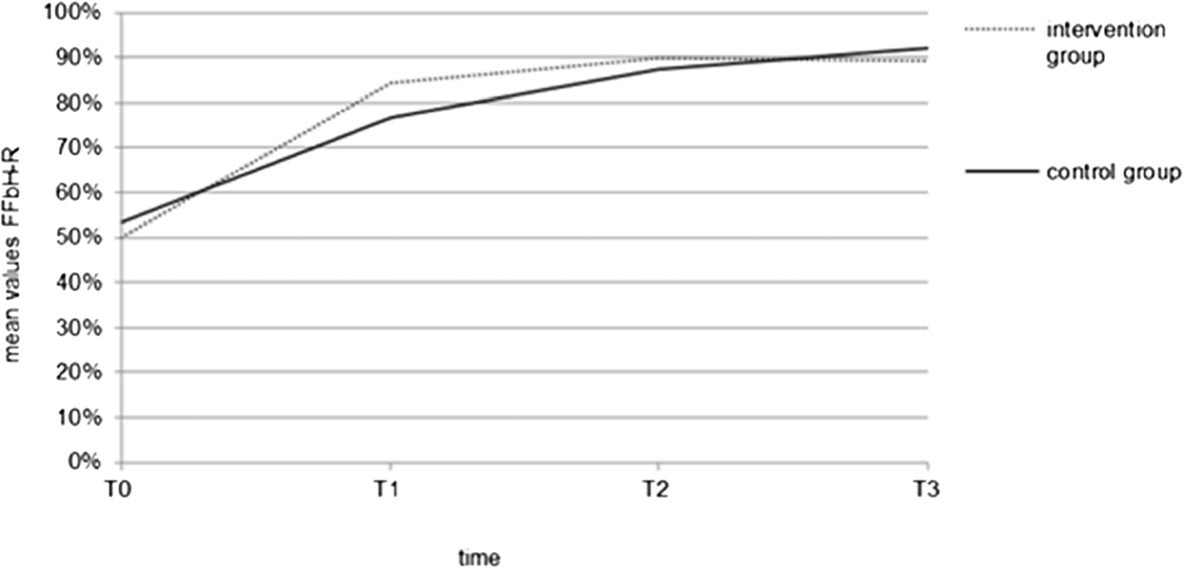
Mean values of FFbH-R (0–100%) from baseline to T3 (12 weeks after treatment)

The mean FFbH-R score increased for both groups during the follow-up period, with the IG showing slightly better functional ability values than the CG by T2 (although without statistical significance). The mean difference between IG and CG in the change in FFbH-R score from T1-T0 was: (7.979 [− 2.382; 18.341] *p* = 0.129). The mean difference between the groups from T0 and T2 was: (− 0.846 [− 8.135; 6.443] *p* = 0.817). However, this slight difference could not be observed after longer periods of time (Fig. 6).

#### Referrals, use of other therapies, days of sick leave

By T2, 24% of the CG and 14% of the IG had been referred to orthopedic specialists. Thirty-six percent of CG and 22% of IG patients used other treatments to alleviate their LBP, most often physiotherapy.

The mean number of days (±SD) of absence from work due to LBP was 3.6 (±9.2) for the CG and 4.0 (±9.5) for IG.

#### Patients’ satisfaction

Both groups of patients were equally satisfied with their GP and the quality of treatment they received.

#### Adverse events

No adverse events were reported during the trial.

## Discussion

The results of the ManRück trial support our hypothesis that MT- techniques in addition to the usual treatment for LBP lead to faster pain reduction and reduced analgesic intake. Moreover, the pain reduction was accompanied by a faster improvement in functional capacity values in the IG, a difference that lasted up to 6 weeks after treatment. Both observations encourage the pursuit of the question of whether MT is a useful option for treating acute non-specific LBP. In general, the ManRück findings confirm the feasibility of this kind of research, while simultaneously indicating improvements which need to be made in the main study. For example, the number of recruited GPs should be increased, and enhanced techniques developed to support their recruitment of patients. The ManRück results showed that the sample size of participating patients must be re-evaluated, as must the time needed for their recruitment. The inclusion and exclusion criteria for patients (e.g., concerning the age limits) should also be critically rethought. Data collection by telephone is preferable to using posted replies, although of course more time-consuming. It would also be beneficial to increase the frequency of the contacts between the study center and the study nurses in the GP’s practices. A further important consideration would be to raise awareness of the study by the use of more target-group adapted flyers, by a higher number of seasonally adapted posters and the use of “new communication channels” (such as electronic messaging on screens in practice waiting rooms) to inform eligible participants of the opportunity of participating in the study.

Recently a randomized controlled trial assessed the effect of interferential current on chronic low back pain, but to our knowledge, only one previous trial involving GPs has considered the effect of MT on acute LBP [15, 22]. Curtis et al. showed a modest improvement in the Roland-Morris functional scale and patient-assessed functional recovery but no effects on pain intensity, sick leave or overall patient satisfaction. However, intervention and control group patients were recruited in parallel by GPs experienced in MT. This may have led to possible selection bias with regard to the patients recruited and the therapy administered. By assessing the usual care of the CG by MT-inexperienced GPs first, then teaching MT-techniques and documenting the results of the add-on MT we avoided this selection bias and so will maintain this concept for the RCT.

To anticipate and avoid expected criticisms concerning the self-limiting character of LBP [9] as raised by B. Walker et al. in a Cochrane review, in the ManRück study changes in pain-intensity were assessed for 1 week immediately following the consultation. Moreover, we restricted MT to mobilization techniques, whereas Curtis et al. used High-Velocity Low Amplitude-thrusts [15]. As the CG patients only visited their GPs once, the IG patients of the ManRück trial also received the add-on-MT only once in order to maximize comparability between the IG and CG. Despite the differences between the two trials, both demonstrated a positive effect of MT in the treatment of LBP in a primary care setting, thus encouraging further investigations.

Because specialists from many different professions performed MT with different approaches, it was difficult to directly compare the results of the MT treatments or the competence and efficiency of the physicians in the trials identified by a systematic literature review. Sometimes osteopaths, chiropractors, orthopedic surgeons, GPs, and physiotherapists applied MT during the same trial [23, 24]. In the ManRück study, all GPs received the same training for the first time, meaning that their initial MT-knowledge at baseline was the same: non-existent. The effects of MT assessed after the training could, therefore, be attributed to the use of the newly learned MT-techniques. This assumption has to be confirmed by further assessments of the quality of the MT that the GPs performed autonomously on the IG. Nevertheless, the methods employed should be evaluated critically at the end of the upcoming RCT in order to endorse this hypothesis. Additionally, the intervention used in our study is not specific and likely to be effective for pain in the sacroiliac joint as well as in the lumbar spine. It remains unclear whether a higher diagnostic accuracy and a more targeted therapy would have led to different results. Controlled trials with a sham therapy currently remain an exception [25].

There is no standardized, generally accepted tool to assess the impact of MT. Available diagnostic tests are controversial [2, 3, 26, 27]. Although some authors insist on a strict separation of NRS and VAS [﻿28﻿], pain intensity was assessed using a combined scale in the ManRück study, which was shown to be both easy for patients to handle and precise for data analysis and calculations.

The ManRück study was designed to investigate the feasibility of an RCT in the GP practice with the aim of reflecting the LBP-concerned population and the real-life practice as closely as possible. For this reason, all patients presenting to their GPs with acute non-specific low back pain were included. This reduced the risk of selection bias from the start and hopefully will maximize the transferability of the results into the daily practice routine. Despite our efforts concerning the study design, a performance bias could not be ruled out with complete certainty, but the successive recruitment of first the CG and then the IG and the positioning of the training sessions between the two recruitment phases ensured that the GPs had no knowledge of MT during CG recruitment and treatment. This minimized a potential source of influence on both the recruitment process and the outcomes of ManRück and will also be taken into account in the RCT.

Due to the lack of randomization in the ManRück study, systematic differences between the CG and the IG group could potentially still occur, such as differences in sex and age. Investigation of the baseline values, however, showed no evidence of such differences between the two groups. To document any influences of potential differences in duration and intensity of the doctor-patient contact on the results of the investigation, both the IG and CG were questioned regarding their satisfaction with their treatment directly after the appointment. Analysis of the data showed no difference on average between the two groups.

The use of a defined standardized MT that predominantly features mobilization techniques may be incompatible with current clinical beliefs that MT should be individualized according to the patient’s overall presentation [29, 30]. Moreover, manipulation is perceived by some as more effective than mobilization techniques [31]. Despite these arguments, we decided to use the standardized mobilization techniques as we aimed to develop an easy and rapidly teachable but effective MT method, likely to produce as few adverse events as possible [32].

The time points at which the outcome parameters were measured were chosen deliberately in order to investigate the immediate effects of MT on acute LBP in the first week after treatment. A 12-week follow-up was used, as, according to the German guidelines, back pain lasting for longer than 12 weeks should be classified as chronic [5]. In the following study, the measurement of pain intensity for at least 12 weeks would also be desirable in order to ascertain the effects of MT given in the acute phase and also to assess the transition from acute to chronic phases of lower back pain.

GPs were asked to register all patients presenting with a backache in a table during the CG and IG recruitment phases, but unfortunately, these data were not collected as intended. The GPs explained that they did not have the time to note down all back-pain patients due to their high number and, instead, concentrated on those fulfilling the criteria for inclusion in the study. According to the GPs, most of the excluded patients had already received other forms of treatment, mostly by physiotherapists or chiropractors. The lacking documentation on excluded patients is an important point to be improved in an RCT to obtain a proper sample description and identify the subgroup population which will benefit most from this kind of intervention.

Finally, we would like to highlight one of the ManRück findings: the reduced analgesic use in the IG. We found this particularly striking, although it had no immediate effect on improving function. It is of special interest for patients with known intolerances to commonly used analgesics such as NSAIDs or acetaminophen, or suffering from side-effects of these medications [33–35] and should certainly be a point of interest in the next study.

### Strength and limitations

ManRück is a study that investigates the use of MT in a GP practice setting and offers a detailed description of the standardized MT technique used, thus ensuring that the study design and the training can be copied and used by any interested party. Moreover, the outcome of the treatments can be contrasted with that of future investigations.

However, this study was not without limitations. Due to the strict inclusion and exclusion criteria, the final sample size was not as high as planned, although recruitment was extended to a total of 23 months, thus reducing the power of the trial. A reason for insufficient recruitment might be the limited financial resources for this preliminary feasibility study. Moreover, there was a change in the study-staff involved in the recruitment, which meant some time passed before an adequate replacement was found. Additionally, in spite of existing estimations of LBP prevalence (point prevalence up to 70% in Germany, including all back pain locations from thoracic- to sacrum spine segments [1] and a mean lifetime prevalence of 38.9% worldwide [36]) a more rigorous assessment regarding the number of patients per practice in Lower Saxony corresponding to our inclusion criteria would certainly help and should be performed before starting a follow-up study. A financial reward for the additional workload of GP and staff (and possibly for the patients) should be budgeted for to improve the recruitment enthusiasm and enhance the response rate.

To verify the promising results of faster pain reduction, future study designs should preferably include a sham intervention. Considering the possibility that the findings of ManRück are due to additional attention, action and time attributed to the patients and not to specific MT-techniques, the assessed outcomes might turn out to be the result of a powerful placebo.

Although the unbalanced distribution of participating patients between the practices was addressed by defining the practices as independent variables in the statistical models, wherever possible this problem should be solved beforehand in upcoming trials. Finally, the trial focused on GPs who were inexperienced in MT, a primary care setting and specifically on adults. For this reason, the effect of MT documented in the ManRück study cannot be generalized for other professionals and is not applicable to younger or older patients or those with specific LBP.

## Conclusions

The pilot ManRück trial explored the possibility of a convenient approach to improve treatment of LBP in a “real life” primary care setting. The employed techniques seemed to be suitable to enrich the usual treatment of LBP in primary care because they apparently reduce the burden of pain. Moreover they may potentially be of use in preventing chronification of LBP and reducing costs of illness and by this worthy of further investigation. The assessment of the effects of MT with a greater sample size, a longer follow-up and a measurement of the acquired competences of GPs in MT by a randomized controlled trial is needed to confirm the findings of the ManRück pilot and to investigate further the possibilities of MT techniques when treating LBP.

## Abbreviations

CG: Control Group
FFbH-R: Hannover functional ability questionnaire-spine
GP: General Practitioner
HVLA: High-velocity low-amplitude
IG: Intervention Group
ITT: Intention to treat
LBP: Lower back pain
MT: Manual Therapy
OMPSQ: Orebrö Musculoskeletal Pain Screening Questionnaire
PD: Patient’s diary
PP: Per Protocol
PSIS: Posterior superior iliac spine
RCT: Randomised Control Trial
SIJ: Sacroiliac joint
VAS-NRS: Visual analog and numeric rating scale
